# Tumor Microenvironment in Gliomas: A Treatment Hurdle or an Opportunity to Grab?

**DOI:** 10.3390/cancers15041042

**Published:** 2023-02-07

**Authors:** Vincenzo Di Nunno, Marta Aprile, Lidia Gatto, Alicia Tosoni, Lucia Ranieri, Stefania Bartolini, Enrico Franceschi

**Affiliations:** 1 Department of Oncology, Azienda Unità Sanitaria Locale (AUSL) Bologna, 40139 Bologna, Italy; 2 Department of Experimental, Diagnostic and Specialty Medicine, University of Bologna, 40136 Bologna, Italy; 3 Nervous System Medical Oncology Department, IRCCS Istituto delle Scienze Neurologiche di Bologna, 40139 Bologna, Italy

**Keywords:** glioblastoma, astrocytoma, oligodendroglioma, microenvironment, immune cells, angiogenesis

## Abstract

**Simple Summary:**

In the present review, we reported the main findings describing tumor-associated microenvironment in patients with IDH mutated and wild-type gliomas. We also focused on the main differences between microenvironment composition reporting data about the microenvironment of pilocytic astrocytomas and IDH wt H3 altered gliomas. This review is finally focused on novel potential treatments targeting the tumor microenvironment.

**Abstract:**

Gliomas are the most frequent central nervous system (CNS) primary tumors. The prognosis and clinical outcomes of these malignancies strongly diverge according to their molecular alterations and range from a few months to decades. The tumor-associated microenvironment involves all cells and connective tissues surrounding tumor cells. The composition of the microenvironment as well as the interactions with associated neoplastic mass, are both variables assuming an increasing interest in these last years. This is mainly because the microenvironment can mediate progression, invasion, dedifferentiation, resistance to treatment, and relapse of primary gliomas. In particular, the tumor microenvironment strongly diverges from isocitrate dehydrogenase (IDH) mutated and wild-type (wt) tumors. Indeed, IDH mutated gliomas often show a lower infiltration of immune cells with reduced angiogenesis as compared to IDH wt gliomas. On the other hand, IDH wt tumors exhibit a strong immune infiltration mediated by several cytokines and chemokines, including CCL2, CCL7, GDNF, CSF-1, GM-CSF, etc. The presence of several factors, including Sox2, Oct4, PD-L1, FAS-L, and TGF β2, also mediate an immune switch toward a regulatory inhibited immune system. Other important interactions are described between IDH wt glioblastoma cells and astrocytes, neurons, and stem cells, while these interactions are less elucidated in IDH-mutated tumors. The possibility of targeting the microenvironment is an intriguing perspective in terms of therapeutic drug development. In this review, we summarized available evidence related to the glioma microenvironment, focusing on differences within different glioma subtypes and on possible therapeutic development.

## 1. Introduction

Gliomas are the most frequent primary central nervous system (CNS) tumors, with an annual incidence of approximately 6 cases per 100,000 individuals worldwide [1].

Histological diagnosis, supported by tissue-based tests (e.g., immunohistochemical), has long been the basis for assessing prognosis and clinical management; nonetheless, the classification of gliomas dramatically changed over the past decade following the advances in molecular analyses.

The fifth edition of the World Health Organization (WHO) Classification of CNS Tumors (WHO CNS 5), published in 2021, enforced the role of molecular parameters, such as altered key genes and proteins, useful in providing diagnostic information and clinically meaningful grading systems [2,3].

The prognosis of patients with gliomas significantly diverges according to tumor histology and molecular features. Indeed, patients with oligodendroglioma and astrocytoma often show several years of long-term overall survival (OS) [4]. On the contrary, glioblastoma (GBM) patients show worse clinical outcomes [4]. Indeed, despite several efforts to improve clinical outcomes, the prognosis of patients with newly diagnosed GBM remains extremely poor (overall survival ranging from 12 to 18 months).

Of note, in WHO CNS 5—GBM comprises only wild-type tumors while *IDH* wild-type (wt) diffuse astrocytic tumors with specific genetic parameters match with GBM diagnosis even in cases that appear histologically lower grade (absence of microvascular proliferation and/or necrosis). Molecular alterations considered to identify molecular GBM are Telomerase Reverse Transcriptase (*TERT*) promoter mutation, Epidermal Growth Factor Receptor (*EGFR*) gene amplification, and combined gain of chromosome 7 and loss of chromosome 10 [2,5].

Beyond genetic and epigenetic data, further elucidation of a tumor’s biological behavior, evolution, and resistance to therapy has recently been enriched with studies on TME (tumor- microenvironment) [6]. The interaction between cancer cells and immune, glial, endothelial cells, neurons, and stem cells composing TME can enhance tumor proliferation and invasiveness, immune suppression, and angiogenesis.

Few studies investigated the composition and role of TME in *IDH*-mutated gliomas, while several data assessing GBM microenvironment composition have been provided. However, a study confirmed that TME composition significantly differs between oligodendrogliomas and astrocytomas [7]. Furthermore, tumors’ grade seems to influence TME composition, especially in terms of macrophage and microglia composition [8]. In these patients, the possibility of manipulating TME composition represents a concrete hope in terms of novel drug development, especially within patients with GBM.

Not surprisingly, the clinical aggressiveness observed in this tumor reflects a complex pattern of molecular alterations, high heterogeneity among tumor cells, and a unique ability to induce phenotypic modifications in other cells, including immune cells, neurons, glial cells, and endothelial/stromal cells [9]. The possibility of targeting these complex interactions could be a promising strategy in terms of novel drug development [10].

In this paper, we analyze current knowledge about microenvironment composition and mechanisms that regulate its interaction with tumor cells, collecting analogies and differences among patients with a diagnosis of astrocytoma, oligodendrogliomas, and GBM (Table 1). Furthermore, we focused our interest on novel possible therapeutic approaches targeting TME.

## 2. IDH Mutated Gliomas

Gliomas harboring *IDH* mutations exhibit better clinical outcomes than wild-type *IDH1/2* tumors. Indeed, the better prognosis is associated with lower histologic grade, *IDH* mutation, and 1p19q codeletion [11,12]. In addition, the type of *IDH* mutation could influence patients’ survival. Indeed, patients with non-canonical *IDH* mutation (*IDH2* or *IDH* mutations other than *IDHR132H*) have a longer survival as compared to patients with canonical *IDHR132H* alteration [15,16,17]. Patients with *IDH*-mutant and 1p19q codeleted gliomas (oligodendrogliomas) have a median OS of about17 years for grade 2 tumors and 11 years for grade 3, longer than survival outcomes reported in patients with *IDH* mutant non-codeleted astrocytomas (mOS of approximately 8–9 years) [11,12,18,19,20,21,22,23,24,25,26,27].

Recent studies support the hypothesis of a progenitor stem cell differentiating towards oligodendrocyte or astrocyte lineage depending on specific genomic alterations. Despite all *IDH*-mutant gliomas seeming to originate from a shared progenitor [7,28], oligodendrogliomas and astrocytomas differ according to morphological aspects, genetic mutations, and TME composition, with subsequent differences in tumor behavior and clinical outcomes.

From a histological point of view, oligodendrogliomas typically show rounded nuclei and a clear perinuclear halo, globally resembling a honeycomb. Astrocytoma is characterized by infiltrating tumor cells with oval to elongated nuclei, varying appearance of cytoplasm, and fine fibrillar processes.

Genomic alterations characterizing astrocytomas include X-linked nuclear protein (*ATRX*) loss (87%), cyclin-dependent kinase inhibitor 2A/2B (*CDKN2A/2B*) homozygous deletion (10%), and *TP53* mutation (94%). Amplification of platelet-derived growth factor receptor (*PDGFR*) genes, *CDKN2A/2B* homozygous deletion, and *PI3K* (phosphoinositide 3-kinase) mutations have been recently associated with worse prognosis in grade 3 astrocytomas [29]. Shifting to oligodendrogliomas (grade 2 and 3), key genetic alterations include Telomerase Reverse Transcriptase (*TERT*) promoter mutation (96%), Capicua Transcriptional Repressor (*CIC*) mutation (62%), Far Upstream Element Binding Protein 1 (*FUBP1*) mutation (29%) and Notch homolog 1(*NOTCH1*) overexpression (31%) [5,12].

Although differences in TME have been described as potential contributors to different biological behavior in *IDH1/2* mutated gliomas, current knowledge on TME composition in these glioma subtypes has yet to be enriched. Herein we will discuss various aspects of TME in the context of *IDH*-mutated gliomas.

### 2.1. Microenvironment in IDH Mutated Gliomas

Interactions between glioma cells and TME contribute to cancer development and progression, influencing prognosis and treatment response. The majority of non-neoplastic cells infiltrating glioma mass are immune cells. Myeloid cells comprising microglia and macrophages represent the predominant immune cell type and are important contributors to disease progression [30,31] (Table 2).

Microglia are CNS-resident myeloid cells migrated into the brain from hematopoietic precursor cells of the yolk sac in early embryogenesis. Conversely, brain macrophages originate from either tissue-resident microglia or monocytes recruited from peripheral circulation [35,36]. Tumor-associated macrophages are plastic and can polarize to either pro-inflammatory (M1) or anti-inflammatory (M2) phenotypes. However, in the glioma microenvironment, they mainly exhibit an immunosuppressive profile. Pro-inflammatory M1 macrophages are involved in anti-tumorigenic effects, while immunosuppressive M2 macrophages are associated with pro-tumorigenic mechanisms that promote tumor growth, angiogenesis, and invasiveness. Different polarization states can coexist in gliomas, and M1/M2 differentiation appears as a continuous spectrum rather than a bimodal scheme [37].

Several studies show a positive correlation between the percentage of microglia/macrophages and glioma grade and a negative correlation with survival. The percentage of myeloid cells in low-grade glioma (LGG) has been estimated at 15–30%, compared with 10–15% in non-neoplastic brain specimens [38]. The degree of macrophage infiltration rather than microglia infiltration in gliomas correlates with tumor grade: data from single-cell RNA-sequencing of human gliomas showed a significant increase in blood-derived macrophages but not an increase in microglia in GBM compared to LGG. Otherwise, astrocytomas have a degree of microglia infiltration higher than both oligodendrogliomas and GBM [39].

Globally, *IDH*-mutated gliomas have been associated with lower infiltration of immune cells in the tumor microenvironment [33,40,41,42,43]. According to evidence of decreased immune cells in *IDH*-mutated glioma, even *IDH*-mutant astrocytoma grade 4, previously known as *IDH* mutant GBM, has been demonstrated to have a lower proportion of tumor-associated microglia and macrophages than *IDH*-wildtype GBM, with predominant M1 polarization [44].

Shifting to the adaptive immune system, *IDH* mutation confers an immunologically quiescent phenotype than wild-type counterparts, with fewer tumor-infiltrating lymphocytes (TILs) and reduced protein expression of programmed death ligand 1 (PD-L1). Consistently, immune suppression in *IDH*-mutant gliomas reflects a reduced expression of interferon-γ (IFN-γ) associated genes and CD8+ T cells (cytotoxic T cells and killer T cells) [41]. Furthermore, it has been hypothesized that high levels of 2-Hydroxyglutarate (2-HG) in *IDH* mutated gliomas indirectly prevent the recruitment of effector T cells (CD8+ cytotoxic and killer T cells, CD4+ helper T cells) by lowering C-X-C motif chemokine ligand 9 (*CXCL9*) and C-X-C motif chemokine ligand 10 (CXCL10) levels, another hypothesis is that 2-HG accumulates and enters T cells altering CD8+ and CD4+ T-cell receptor (TCR) signaling pathways, thus resulting in impaired T cell anti-tumor immunity [33]. The 2-HG is secreted by tumor cells and imported by CD8+ T cytotoxic and CD4+ T helper cells by a sodium-dependent dicarboxylate transport system and inhibits TCR by interfering with the TCR—ATP dependent signaling and a polyamine biosynthesis pathway [42].

Recently, a deep analysis of immune gene profiles of LGG patients from The Cancer Genome Atlas has led to the identification of three distinct immune subtypes—Im1, Im2, and Im3—which differ in lymphocyte signatures, genetic alterations, and clinical outcomes. Of note, Im1 and Im2 were enriched in *IDH1* mutation and had lower immune infiltrate. Im1 was characterized by higher infiltration of CD8+ cytotoxic T cells, Th17, and mast cells; this profile was enriched in *IDH1*, 1p/19q codeletion, *CIC*, *FUBP1*, and *NOTCH1* mutations. Im2 showed a high lymphocytic infiltrate, high M2 macrophage content, and checkpoint gene expression, indicating an immune-hot but immune-suppressive TME. Im2 was enriched in mutations in driver genes, such as *PTEN*, *EGFR*, and *NF1*. Finally, Im3 displayed higher levels of T CD4+ helper cells, antigen-presenting cells, and macrophages; this subtype was enriched in *IDH1*, *ATRX*, and *TP53* mutations [45].

Beyond immune suppression, the accumulation of 2-HG due to *IDH* mutation determines epigenetic changes that modify the tumor microenvironment by inhibiting angiogenesis. Of note, 2-HG can stimulate prolyl-hydroxylases such as egl-9 family hypoxia-inducible factor 1 (*EGLN*) which inhibits hypoxia-inducible factor 1, subunit alpha (HIF1α) [34].

Reduced HIF-1α levels in *IDH* mutant gliomas reflect epigenetic silencing of glycolytic switch–related genes; on the contrary, *IDH* wt gliomas show a glycolytic phenotype. As a result, in *IDH* wt gliomas, TME is characterized by higher acidity and higher hypoxia than in *IDH* mutant gliomas. In this way, metabolic reprogramming could affect tumor aggressiveness. *IDH* mutant astrocytoma rather than oligodendroglioma has been associated with the acquisition of a glycolytic phenotype, explaining the worse outcomes [46].

Interactions between neoplastic cells and neurons and normal glial cells in *IDH* mutated gliomas are currently less explored than immune microenvironment. Neurons and glial cells are supposed to display marked plasticity during tumor progression, which could also be the reason for long pre-symptomatic periods in LGG. In addition, there is increasing data that excitatory neurotransmitter glutamate secreted by glioma cells induces hyperexcitability and excitotoxicity of peritumoral neurons playing a critical role in the growth and spread of glioma cells. However, most evidence derives from studies on high-grade gliomas [47].

### 2.2. Differences in Astrocytoma and Oligodendroglioma Tumor Microenvironment

Differences in tumor microenvironment composition among astrocytoma and oligodendroglioma mainly emerge from studies on the immune microenvironment, as this is currently the most explored field. Available evidence suggests dissimilarities in innate immune cell populations among *IDH*-mutated gliomas. Focusing on the microglia/macrophage population, Venteicher et al. identified two different inflammatory signatures and provided a relationship with tumor grade and subtype. In astrocytoma, microenvironment expression reflects a macrophage signature; oligodendrogliomas, instead, reflect a microglia signature. Microglia signature was associated with a peculiar expression of C-X-C motif chemokine receptor 1 (CX3CR1), Purigenic Receptor P2Y12 (P2RY12), and Purigenic Receptor P2Y13 (P2RY13), the second signature, identified as macrophage signature, was characterized by high expression of CD163, Transforming Growth Factor-β1 (TGF-β1) and coagulation factor XIII A chain (F13A1). Higher-grade tumors were associated with more macrophage-like expression states. Macrophage signature rather than microglia signature influenced angiogenesis and alterations of the blood–brain barrier. Mechanisms that determine macrophage-like and microglia-like expression states are still poorly characterized in LGG. However, genetic alterations have been proposed to affect microglia/macrophage signature balance [7].

While several publications describe increasing microglia/macrophages levels in diffuse gliomas according to the WHO grade, a study assessing the immunohistochemical expression of selected microglia and macrophage markers in grade 1–4 gliomas surprisingly found a higher pan-macrophage markers expression and a marked M2 polarization in pilocytic astrocytoma (WHO grade 1) compared to diffuse astrocytomas and GBM [48].

Despite astrocytoma and oligodendroglioma showing similar compositions in terms of immune cells, when assessing tumor-associated lymphocytes in LGGs, astrocytomas display a more immunosuppressive local microenvironment, with increased percentages of PD-1 + CD8+ cytotoxic T cells, T-cell immunoglobulin and mucin-domain-containing-3 + (TIM-3) CD4+ T cell subpopulations and regulatory CD4+ T cells (Tregs) [8].

In another study comparing data on LGGs from TGCA and Chinese Glioma Genome Atlas (CGCA), 1p/19q codeletion has been associated with lower infiltrating levels of immune cells and lower expression of immune checkpoint genes compared to 1p/19q non-codeleted cohorts. Since chromosome 1p or 19q genes host several genes involved in inflammatory pathways, such as *TGFB1*, *JAK1*, and *CSF1*, 1p/19q codeletion can result in an altered infiltrating level of immune cells and expression of immune checkpoint genes [49].

Peculiar evidence on TME composition in LGG also comes from the study of tumor networks, an attractive research field. With regards to LGG, particularly oligodendrogliomas, growth-associated protein 43 (GAP43) regulates microtube outgrowth and is linked to tumor cells’ invasiveness and ability to recolonize the surgical site. A codeletion of both chromosomal arms in oligodendrogliomas leads to less microtube formation and fewer interconnected tumor cells [50].

### 2.3. Pilocytic Astrocytoma Microenvironments

Pilocytic astrocytoma (PA) is classified as grade 1 lesions without significant proliferative potential and often without relapse after total curative resection [2]. The term pilocytic refers to elongated projections visible after staging of glial fibrillary acidic protein in the cytoskeleton of these cells [51,52]. The Rosenthal fibers are mainly composed of crystalline α-β, hyaline structures without glial fibrillary acidic protein immunoreactivity observable after thermic or oxidative shock [51,52]. Regarding molecular alterations, childhood Pas often show alterations in the *MAPK* (mitogen-activated protein kinase) pathways, including mainly *BRAF* mutations [53]. Of note, the fusion between *BRAF* and *KIAA1549* gene is also found in the majority of PAs [2,52].

There are very few studies assessing the composition of PAs microenvironment. In 2011, Yang I et al. compared immune cells’ microenvironment composition of glioblastoma and PAs showing that GBM specimens had a significantly higher percentage of perivascular CD8+ T cell, perivascular and intratumoral CD-56+ T cell (Natural Killer T cells), and macrophages while there was no difference between CD3+ and CD 20+ T cells [54]. A more recent study assessed the tumor methylome of PAs by generating whole genome bisulfite sequence (WGBS) data from 9 PA patients [55]. The authors identified that the basic leucine zipper (bZIP) transcription factors were an important positive regulator of the immune response [55]. To date, no studies investigated differences between *IDH*-mutated gliomas and PAs microenvironment composition.

## 3. IDH wt Gliomas

*IDH* wt gliomas are a heterogenous class of tumors, both on histological and molecular levels. Glioblastoma is histologically defined as an infiltrating astrocytic glioma with microvascular proliferation or necrosis characterized by a lack of mutations in *IDH1*, *IDH2*, and histone *H3* genes. The WHO CNS 5 2021 classification defined as molecular GBM also diffuse glioma with *TERT* mutation and/or *EGFR* amplification and/or +7/−10 chromosome deletion [14].

Among *IDH* wt gliomas, the 2021 WHO classification recognizes novel tumor entities characterized by histone 3 gene alterations [2]. The diffuse midline glioma is characterized by *H3* alterations, often resulting in lysine-to-methionine substitution at position 27 of histones 3.1 and 3.3 (*H3K27*) [56]. Other molecular alterations leading to the development of diffuse midline glioma have been described, including *EZH* inhibitor protein (*EZHIP*) mutations [56].

### Microenvironment in IDH wt Gliomas

**Figure 1:**
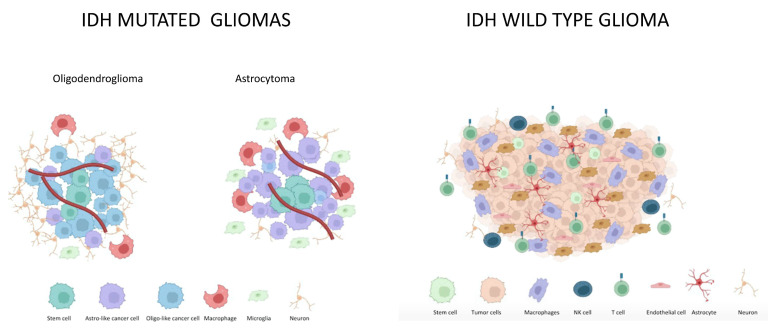
The tumor-associated microenvironment in IDH-mutated and IDH-wt gliomas.

Most studies aiming to describe TME in *IDH* wt gliomas effectively assessed the TME composition of GBM (Figure 1).

In recent years, single-cell RNA-sequencing (scRNAseq) studies have acquired a higher relevance in the description of TME. Additionally, these studies uncover inter-patient to intra-tumoral heterogeneity and come up with molecular information at a single-cell level. With the scRNA seq technique, DNA sequencing and data analyses are performed on genetic material derived from individual cells, physically isolated from each other. In contrast with bulk methods, which provide an average estimation of gene expression across all cells in a sample, scRNAseq allows the description of cellular subpopulations, including rare cell phenotypes [57].

Reasons for dismal prognosis and lack of novel effective therapies in GBM are closely related to biological features of this tumor: intra- and inter-tumoral heterogeneity, presence of GBM stem cells (GSCs), and the interplay between cancer cells and TME. Glioblastoma cells communicate with each other and with the surrounding environment through various mechanisms. Routes of communication include gap junctions, extracellular vesicles, nanotubes, and microtubes. These mechanisms allow the translocation of genetic elements, proteins, and other metabolites. One of the emerging research fields focuses on neuron-to-brain tumor synaptic communication. This kind of synapse has been described in low-grade and anaplastic astrocytomas, GBMs, and diffuse intrinsic pontine gliomas [58,59]. Synaptic communication is facilitated by specific neurotransmitter receptors on glioma cells located in thin membrane tubes, called microtubes, similar to axonal and dendritic neuronal outgrowths. Postsynaptic currents are mainly mediated by glutamate receptors of the α-amino-3-hydroxy-5-methyl-4-isoxazole propionic acid (AMPA) subtype, in particular calcium-permeable AMPA receptors. Depolarization of the tumor cell membrane due to short calcium transients activate downstream pathways. Preclinical studies show a relationship between the activation of glutamatergic neuron-to-glioma synapses and the invasiveness of glioma cells, in particular in the initial stages of tumor progression [58]. Recently, Venkataramani et al. reported that GBM is composed of both tumor cells that form a network interconnected by microtubes and by other subpopulations that appear unconnected. This latter subpopulation transcriptionally resembles neuronal-and neural-progenitor-like cell states and seems to receive neuronal synaptic input and drive brain invasion [60].

Paracrine signaling through soluble factors such as brain-derived neurotrophic factor (BDNF), 78 kDa glucose-regulated protein (GRP78), and neuroligin-3 (NLGN-3) is another route of communication in brain tumor networks. NLGN-3 is released by neurons and oligodendrocyte precursor cells and activated after cleavage by the metalloproteinase ADAM10. It binds on glioma cells leading to PI3K-mTOR signaling activation and promoting tumor growth [50].

Apart from synaptic and paracrine, intercellular communication systems through RNA transfer is one of the most challenging areas of research. A study investigating the spectrum of cancer-derived extracellular RNAs (exRNAs) by tumor-derived cells from GBM patients showed exRNA is enriched in small non-coding RNAs, such as microRNAs (miRNAs) in exosomes, and tRNA and Y RNA fragments in extracellular vesicles (EVs) and ribonucleoproteins (RNPs). The most common extracellular tRNA fragments are produced by angiogenin, a multifunctional ribonuclease that regulates angiogenesis, cell proliferation of cancer cells, neuronal survival, and stress response. Angiogenin is upregulated in GBM and, in particular, in exosomes [61]. Another class of non-coding exRNAs, evolutionarily conserved molecules involved in many cellular functions, is still poorly described. Finally, miRNAs represent the most studied class of exRNA. In particular, microRNA-10b (miR-10b) has been reported as the most upregulated miRNA in GBM across all subtypes and is undetectable in normal brain tissues, thus appearing as a potential therapeutic target. MiRNA-10b seems to promote cell cycle progression (S-phase and mitotic transitions), migration, invasion, and survival of glioma cells. Analysis of GBM tumors using TCGA suggested that miR-10b regulates E2F1-mediated transcription in GBM [62].

Regarding interactions between GBM cells and non-tumor cell populations, immune cells are the most studied component. In particular, tumor-associated microglia and macrophages (TAMs) account for about 30–50% of the tumor mass. Globally, the percentage of TAMs has been related to higher glioma grades and worse survival. *IDH*-wildtype GBM and metastatic brain tumors are characterized by the highest influx of macrophages. While in the early phases of tumor growth, infiltrating cells are mainly represented by microglia, in advanced phases, macrophages, and myeloid cells are the most abundant ones. The infiltration of peripheral immune cells, in particular bone marrow-derived monocytes and macrophages, is favored by the disruption of the blood–brain barrier determined by GBM itself and the release of cytokines and chemokines by glioma cells such as CC-chemokine ligand 2 (CCL2), CCL7, glial cell line-derived neurotrophic factor (GDNF), colony-stimulating factor-1 (CSF-1), granulocyte-macrophage colony-stimulating factor (GM-CSF), hepatocyte growth factor (HGF), stroma cell-derived factor 1 (SDF-1). Once recruited, monocytes and macrophages can acquire different phenotypes, ranging from M1 (tumor-suppressive) and M2 (tumor-supportive) phenotypes. Indeed, GBM—released factors drive the activation state of these cells. Overall, GBM cells suppress the immune response against tumors, shifting the phenotype of surrounding immune cells toward an immune-suppressive, tumor-supportive state. Subsequently, TAMs contribute to tumor proliferation, supporting ECM remodeling and angiogenesis. Neutrophils and mast cells are recruited by GBM cells and are involved in tumor growth in GBM [63,64,65].

Besides innate immune cells, also an adaptive immune response to brain tumors has been described. Experiments conducted in mice models suggested that antigen-presenting cells (APCs) move from the CNS to deep cervical lymph nodes, where they present brain tumor antigens to T cells. Lymphatic vessels in the meninges constitute a direct drainage to the cervical lymph nodes, and microvascular changes near the tumor allow immune cells to enter CNS. Vascular abnormalities are a common hallmark of GBM, with hypermediated, permeable vessels and highly elevated levels of vascular endothelial growth factor (VEGF) in TME.

Although the ability of immune cells to enter the CNS, various immune escape mechanisms make the GBM immune microenvironment “cold”. The TGFβ2 is a cytokine isolated for the first time in GBM which contributes to T cell exhaustion through various mechanisms such as suppression of IL-2 (Interleukin-2) dependent T cell survival and expression of co-inhibitory receptors on CD4+ T helper and CD8+ cytotoxic T cells. Concurrently, GBM cells show membrane-bound factors such as Fas antigen ligand (FAS-L) and programmed cell death ligand 1 (PD-L1), which are well-known co-inhibitory molecules. More recently, SRY-Box Transcription factor 2 (Sox 2) and octamer-binding transcription factor 4 (Oct4) have been related to the downregulation of Th1 response, stimulation of Treg, and expression of co-inhibitory molecules. Indeed, the co-expression of Oct4/Sox2 inhibits the expression of the C-C Motif Chemokine ligand 5 (CCL5) and C-X-C Motif Chemokine ligand 9, 10 and 11 (CXCL 9,10 and 11), which mediate CD8+ T cell attraction against tumor cells [66]. Furthermore, Sox2/Oct4 mediates the expression of interleukin 8 and 6, which, together with the signal peptide peptidase, mediate the shift of macrophage phenotype toward an immune-regulatory profile inhibiting immune response against tumor cells [66].

These effects are mediated by a family of proteins named Bromodomain and extra terminal motif (BET) proteins, which have lately been explored as potential pharmacological targets [66,67].

Increasing evidence seems to confirm that prostaglandin E_2_ (PGE2) is abundant in the GBM microenvironment and mediates tumor invasion and progression, while cyclooxygenase (COX) inhibition is associated with reduced proliferation and tumor cell migration in vitro [68,69,70,71,72]. Some studies assessed the role of COX inhibitors in GBM but, to date, none of them have shown to significantly improve survival or other patients’ clinical outcomes [73,74,75,76,77].

Among non-Immune cellular components, stem cells associated with GBM (GSCs) have been recently studied, given their importance in the natural history of this tumor. GSCs are located in a specific biological niche, perivascular niche, and can differentiate either into cancer cells or normal cells. Little is known about pro-differentiative signals determined by TME. However, a recent study provided evidence that differentiation towards oligodendrocyte lineage is sustained by tumor-infiltrated white matter in vivo. Concurrently, other studies have demonstrated the ability of GSCs to differentiate into endothelial cells and pericytes. Moreover, GSCs can directly respond to hypoxia-stimulating VEGF—mediated neoangiogenesis [78,79]. Astrocytes greatly contribute to neoangiogenesis, enhancing tumor growth through the release of cytokines (such as TGFβ and Il-6) and growth factors. Additionally, in vitro studies demonstrated their role in resistance to chemotherapy. The crosstalk between glioma cells and tumor-infiltrating astrocytes can lead to the transformation of astrocytes into neoplastic glioma cells [32]. As regards oligodendrocytes, instead, they seem to exhibit an inhibitory function against GBM through the activation of the WNT inhibitory pathway (Table 2).

## 4. Differences in IDH-Mutated and IDH-wt Tumor Microenvironment

The tumor microenvironment of *IDH*-wt tumors is composed of a higher percentage of immune cells as compared to *IDH*-mutated gliomas [7,9] (Table 2). Indeed, *IDH*-mutated tumors present a lower infiltration of microglia and macrophages as compared to *IDH*-wt tumors (macrophage signature predominant in astrocytoma, while microglia signature is most represented in oligodendroglioma) [7,8,9]. The percentage of tumor-associated microglia and macrophages is correlated to tumor grade and is higher in glioblastoma, in which macrophages drive extracellular matrix remodeling and angiogenesis [9]. The 2-Hydroxyglutarate inhibits angiogenesis in *IDH*-mutated tumors [33], while the neo-vessels development is stimulated by macrophages and astrocytes recruited around *IDH*-wt cells [34]. In *IDH*-wt tumors, the interactions between neoplastic and normal cells (including gap junctions, extracellular vesicles, nanotubes, microtubes, paracrine signaling, and extracellular RNA) play a critical role in stimulating the neoplastic transformation of normal cells (astrocytes) [32], neoangiogenesis, tumor invasion, and progression [9]. These same interactions have not been described in IDH-mutated tumors. Within *IDH*-mutated tumors, astrocytoma rather than oligodendrogliomas often presents an increased percentage of PD-1+ and TIM-3+ T cells [8]. In glioblastomas, several factors (TGF β2, FAS-L, PD-L1, Sox2, Oct4) secreted by the same innate immune cells contribute to developing a T cell-exhaustion phenotype [9]. To date, there are data evaluating interactions between neurons and *IDH*-mutated tumor cells. On the contrary, it seems that these same interactions play a crucial role in *IDH*-wt tumor cells. Indeed, glutaminergic neurons seem to mediate glioma invasiveness and progression [9].

## 5. Future Perspectives

To date, few treatment improvements have been documented for patients with gliomas. Target therapies, including *BRAF* and *NTRK* inhibitors, showed promising clinical efficacy in glioma patients with these molecular alterations [53,80,81,82]. The multi-tyrosine kinase inhibitor regorafenib, compared to lomustine, improved survival and other clinical outcomes in patients with GBM in phase II randomized clinical trials. In a small percentage of cases, glioma patients harbor fibroblast growth factor receptor (FGFR) mutations which make them targetable from specific FGFR inhibitors [83], including infigratinib (NCT04424966, NCT05222165) and pemigatinib (NCT05267106).

Recent research on novel treatments for gliomas comes from insights into the biology of these tumors. Potential targets include the aforementioned routes of communication between glioma cells and TME, as well as molecules directly involved in tumor-supportive mechanisms.

Inhibitors of molecules involved in neuron-tumor and tumor-tumor networks have been assessed in preclinical studies and early-phase clinical studies. Recent research details glutamatergic neuro-glioma synapses as a target for glioma treatment. Neuron-glioma glutamatergic synaptic transmission can be blocked by AMPA receptor inhibitors such as talampanel or perampanel [84].

Among attractive targets, the immune environment appears as the most promising field both in IDH mutated and in IDHwt gliomas. Given the striking benefits reported with checkpoint inhibitors in the treatment of various solid tumors, great interest has been placed in this kind of therapy. Unfortunately, several trials of checkpoint inhibitors have not been demonstrated to improve clinical outcomes in low-grade and high-grade gliomas. This could be addressed to the typical immune suppressive signature and the heterogenous composition of these tumors, with a variable expression of tumor-specific antigens. Recently, results from phase III Checkmate548 showed that adding Nivolumab to radiotherapy plus temozolomide did not improve survival in patients with newly diagnosed GBM with methylated or indeterminate MGMT promoter [85]. A phase III study is evaluating the efficacy and safety of nivolumab administered alone versus bevacizumab in patients with recurrent GBM and the safety and tolerability of nivolumab alone or combination with ipilimumab in recurrent GBM previously treated with different lines of therapy (NCT02017717).

Increasing data suggest that Poly (ADP-ribose) Polymerase (PARP) inhibitors could be active in patients with BRCA and homologous recombination deficiency (HRD) [86]; thus, the combination between the PARP inhibitor Olaparib, the PD-1 inhibitor pembrolizumab and temozolomide is currently under investigation in phase II clinical trial (NCT05188508). At the same time, niraparib is currently under investigation in patients with recurrent *IDH* wt and mutated gliomas (NCT05297864).

Selumetinib is an inhibitor of the mitogen-activated protein kinase (*MEK*) which showed promising clinical efficacy on neurofibromatosis-1 (NF1) associated gliomas [86,87,88,89]. Two trials are investigating this agent in patients with glioma (NCT03871257, NCT04166409).

In IDH wt H3 altered gliomas, there is a novel promising agent targeting the Dopamine receptor 2(DRD2) as well as the proteolytic subunit of mitochondrial protease Clp (CIpP) [90,91,92]. This agent showed promising clinical activity, and other trials are currently investigating these compounds in patients with H3-altered IDH wt gliomas [13] (NCT05580562, NCT04541082).

Of interest, novel trials are assessing combination strategies to target different immune escape mechanisms simultaneously. Some trials are exploring the safety and efficacy of combining chemotherapy, anti- PD1 inhibitors, or radiotherapy with inhibition of other immune checkpoints, such as lymphocyte activation gene 3 (LAG 3) and indoleamine 2,3-dioxygenase (IDO1) in GBM. Recently, killer cell lectin-like receptor subfamily B member 1 (KLRB1), encoding the T cell receptor CD161, has been identified in cytotoxic T cells in gliomas and has been proposed as a novel potential target for immunotherapy in diffuse gliomas [93,94].

Furthermore, immunological approaches have recently been enriched with novel tools. Chimeric antigen receptor T cells (CAR-T) and chimeric antigen receptor macrophages (CAR-M) [95] are recombined cells obtained from patients and activated in vitro against specific antigens. GBM-specific cell surface antigens studied as potentially suitable targets for CAR T cells include the B7 homolog 3 immunoregulatory protein (B7-H3), epidermal growth factor receptor VIII (EGFR VIII), HER2, IL-13 receptor α chain 2, disialoganglioside GD2. Recently, a preclinical study using patient-derived GBM cells confirmed GD2 antigen as a potential target for the CAR T strategy [96]. Of note, GD 2 is highly expressed in GBM cells while representing 1–2% of the total amount of gangliosides in the normal central nervous system. Data from a phase I clinical trial in H3K27M-mutated glioma recently reported clinical and radiographic improvement in three of four patients treated with GD2 CAR T cells [97].

Additionally, peptide vaccines and dendritic cell vaccines targeting H3K27M mutation showed promising results in preclinical studies.

Vaccine injection has been studied in IDH-mutated gliomas, both in preclinical and clinical trials. A first-in-human phase I trial with IDH1(R132H) specific peptide vaccine, named NOA16 trial, was conducted in newly diagnosed grade 3 and 4 astrocytomas carrying the specific mutation, with 93.3% of patients presenting an immune response and good safety profile [98]. IDH inhibition through molecules such as ivosidenib and vorasidenib improved survival outcomes in early-phase trials. However, the relationship between IDH inhibition and modification of TME must be clarified [99].

Immunotherapy through vaccine injection is currently under investigation in several phase 2 and 3 trials in GBM. Among these, many studies are exploring treatment with dendritic cell-vaccines (NCT03548571, NCT04277221, NCT04888611, NCT03688178). These vaccines use DCs to deliver antigens and stimulate host immune activation against the tumor. Recently, in a phase III nonrandomized controlled trial of 331 patients, adding DCVax-L (lysate-loaded dendritic cell vaccination) to the standard of care demonstrated a clinically and statistically meaningful improvement in median OS in patients with either newly diagnosed or recurrent GBM [100]. The conjugate vaccine SurVaxM adopts surviving as an antigen to stimulate a CD8 lymphocyte to mediate response against the tumor. In a phase IIA study, this vaccine has been proposed after surgery and radiotherapy with concurrent temozolomide in patients with newly diagnosed GBM. In this cohort, the vaccine showed promising clinical efficacy in both methylated and unmethylated patients achieving a PFS and OS of 11.4 and 25.9 months in the overall population [101]. In a phase 3 study, DSP-7888, an immunotherapeutic cancer vaccine derived from the Wilms’ tumor gene 1 (WT1) protein, is under evaluation in combination with Bevacizumab versus Bevacizumab alone in patients with recurrent or progressive GBM (WIZARD 201G, NCT03149003). (Table 3).

Some phase II trials are focusing on targeting specific cytokines in TME: L19TNF, a fully human fusion protein consisting of human tumor necrosis factor (TNF)-α fused to the L19 antibody, induces apoptosis or necrosis in target cells, stimulates inflammation and immunity (NCT04573192, NCT04443010), olaptesed pegol (NCT04121455) specifically binds to SDF-1 thereby preventing the binding to its receptors CXCR4 and CXCR7.

The possibility of shifting TME composition favoring tumor regression and immune response has led to the development of interesting novel approaches. It has been demonstrated that immune-activating cytokines such as interferon α (INF α) and interleukin 12 can modify immune-cells composition on TME, favoring and restoring an immune response against tumors [104,105,106]. Unfortunately, systemic administration of these cytokines is associated with significant and limiting side effects. Gene-and cell-based delivery of cytokines may overcome these limitations. Tie2 (Tek tyrosine kinase receptor)-expressing cells are a unique class of monocytes due to their ability to stimulate paracrine pathways resulting in angiogenesis and tumor growth [107,108,109,110]. These cells have been assessed as a candidate for cell-based delivery of cytokines in preclinical and early clinical studies. A phase I/II study is currently assessing tamferon: an autologous hematopoietic progenitor cell expressing TIE2 and exposed to a viral vector (lentiviral vector) encoding INF α gene (NCT03866109).

Targeting angiogenesis is a current investigational field, with the humanized monoclonal anti-VEGF antibody bevacizumab being the most studied molecule and the only FDA-approved treatment of recurrent GBM. Bevacizumab is, however, burdened by no impact on overall survival due to resistance mechanisms determined by marked hypoxia inside the tumor. Retrospective studies in GBM showed administration of inhibitors of the renin-angiotensin system during standard treatment reduced peritumoral edema, lowered dosages of steroids, and improved survival [111].

Ongoing clinical trials are testing bevacizumab in combination with additional agents, such as immune checkpoint inhibitors and radiotherapy, in various settings. (NCT03452579, NCT03661723, NCT03743662). (Table 3 and Table 4). Apatinib is another specific VEGFR2 inhibitor that is currently under investigation in combination with temozolomide in patients with newly diagnosed high-grade gliomas (NCT03741244).

## 6. Conclusions

In recent years molecular characterization of gliomas has provided more accurate tools to differentiate glioma subtypes, thus explaining differences in natural history and response to treatments. Studies on tumor microenvironments have revealed further mechanisms useful to understand the biology of these tumors. Even if the majority of studies in the literature focused on GBM, there is also growing evidence of IDH-mutated astrocytoma and oligodendroglioma. The most promising field deals with the immune microenvironment, reporting a relationship between higher tumor grade and marked immunosuppressive signatures. IDH mutated gliomas have been associated with lower infiltration of immune cells than IDH wt gliomas. Interactions of glioma cells along with immune cells appear to be a challenging target for the development of new therapeutic strategies, such as CAR-T and CAR-M. Vaccines recently showed their clinical efficacy also in GBM patients in early settings of treatment. This enhances the importance of the immune-associated microenvironment and justifies further efforts toward the development of trials investigating agents able to restore immune response against tumors.

## Figures and Tables

**Table 1:** Clinical, morphological, and genomic/epigenetic characteristics of gliomas.

	Oligodendroglioma	Astrocytoma	H3—Altered Gliomas	Glioblastoma IDH wt
Morphology	- rounded nuclei - clear perinuclear halo [11,12] - globally resembling a honeycomb [11,12].	- oval to elongated nuclei [11,12] - varying appearance of cytoplasm [11,12]; - fine fibrillar processes.	- astrocytoma-like neoplastic cells; - microvascular proliferation and necrosis possible [13]	- microvascular proliferation; - necrosis [14].
Genomic and epigenetic alterations	- IDH1/2 - 1p19q codeletion - G-CIMP; - TERT (96%); - CIC (62%); - FUBP1 (29%); - NOTCH1 (31%) [11,12].	- IDH1/2 mutation - ATRX loss (87%); - G-CIMP; - CDKN2A/2B (10%) - TP53 (94%) [11,12].	- H3 alterations; - EZHIP - TP53 (70%); - ATRX (30%); - PDGFRA (45%); - Loss of H3 trimethylation [13].	- TERT (70%); - EGFR Amplification (40%); - +7/−10 Chromosome; - TP53 (20%) [14].
Prognosis	8–17 years [11,12]	6–12 years [11,12].	9–24 months [11,12]	9–24 months [11,12]

ATRX: X linked nuclear protein; CDKN2A/2B: cyclin-dependent kinase inhibitor 2A/2B homozygous deletion; CIC: Capicua Transcriptional Repressor; FUBP1: Far Upstream Element Binding Protein 1; G-CIMP: cytosine-phosphate-guanine (CpG) island methylator phenotype; EZHIP: Enhancer of Zest Homologs Inhibitory Protein, H3: Histone 3, NOTCH: Notch homolog 1; IDH: Isocitrate dehydrogenase; PDGFRA: Platelet-derived growth factor receptor A; TERT: Telomerase Reverse Transcriptase.

**Table 2:** The tumor-associated microenvironment in IDH-mutated and IDH-wt gliomas [7,8,9,32,33,34].

	IDH Mutated Gliomas		IDH Wildtype Gliomas
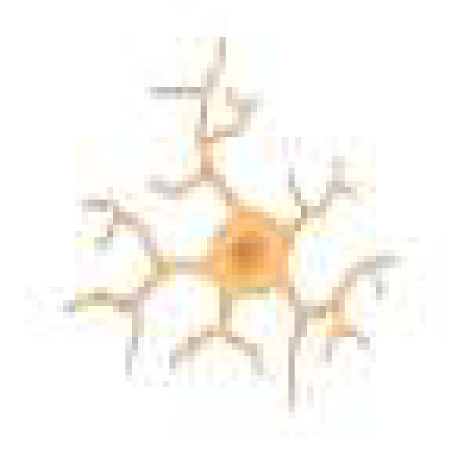	Lower infiltration of immune cells than IDH wt gliomas, comprising lower microglia and macrophages percentage.	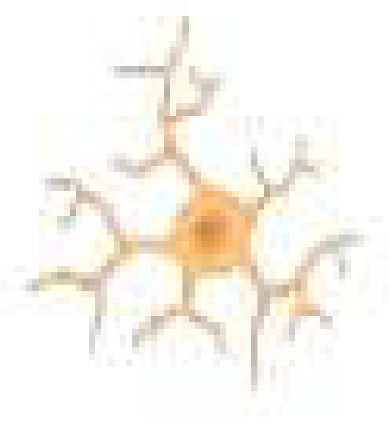	Higher percentage of tumor associated microglia and macrophages is related to higher glioma grade, driving ECM remodelling and angiogenesis.
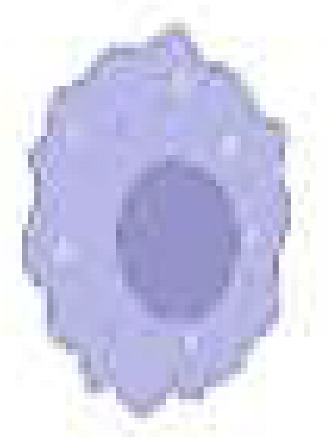	TME in astrocytomas reflects a predominant macrophage signature. In oligodendroglioma myeloid immune cells show mainly microglia expression states [7,8]. The 2-HG in IDH mutated gliomas interferes with recruitment and function of T cells [33].	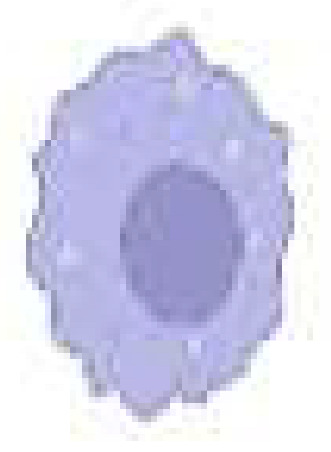	Myeloid cells in IDH wt gliomas reflect a predominant machrophage signature [33]. Infiltration by immune cells is favored by cytochines and chemokines from glioma cells (CCL2, CCL7, GDNF, CSF-1, GM-CSF, HGF, SDF-1). Also neutrophils and mast cells are recruited by GBM [32,33,34].
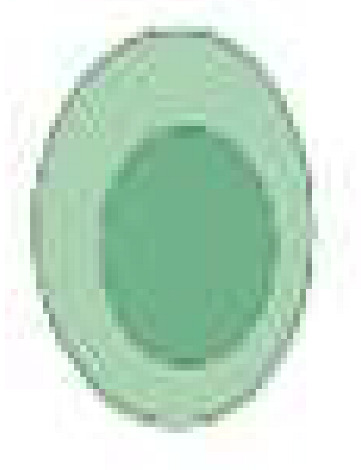	Astrocytoma rather than oligodendroglioma display increased percentages of PD-1+ CD8+ Tcells, TIM-3+CD4+T cells and T regulatory cells.	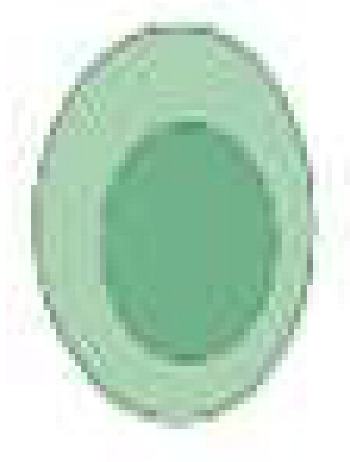	Several factors contribute to T cell-exhaustion (TGF β2, FAS-L, PD-L1, Sox2, Oct4).
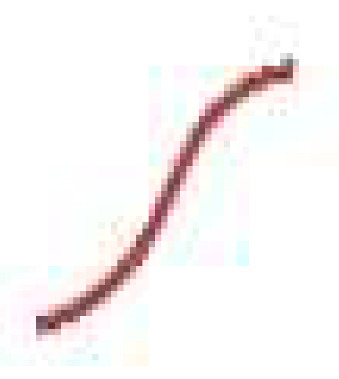	2-HG is supposed to inhibit angiogenesis. Additionally through reduced HIF-1α levels, it inhibits glycolytic switch-related genes, tipically expressed in IDH wt subtypes [7,8,9,33].	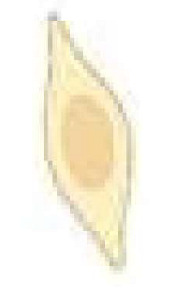	Stem cells located in the perivascular niche can differentiate either into cancer cells or normal cells.
	Interactions with normal glial cells and neurons, interactions with stem cells need to be elucidate	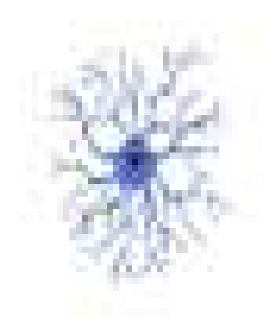	Astrocytes contribute to neoangiogenesis in GBM. They can undergo neoplastic transformation [32,33,34].
		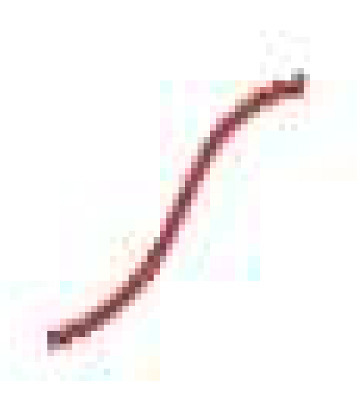	Interactions between cells involve multiple routes of communication (gap junctions, extracellular vescicles, nanotubes, microtube, paracrine signaling, extracellular RNA).
			Glutamatergic neutron to brain tumorsynapses are involved in glioma invasiveness.
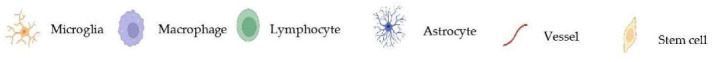			

IDH, Isocitrate dehydrogenase; TME, tumor microenvironment; 2-HG, 2-hydroxyglutarate; PD-1, Programmed death-1; TIM, T-cell immunoglobulin and mucin-domain-containing-3; ECM, extracellular matrix; CCL2, CC-chemokine ligand 2; CCL7, CC-chemokine ligand 7; GDNF, glial cell line-derived neurotrophic factor; CSF-1, colony-stimulating factor-1; GM-CSF, granulocyte-macrophage colony-stimulating factor, HGF, hepatocyte growth factor; SDF-1, stroma cell-derived factor 1; FAS-L, Fas antigen ligand; Sox2, SRY-Box Transcription factor 2; Oct4, octamer-binding transcription factor 4.

**Table 3:** Ongoing phase III trials targeting tumor microenvironment in glioblastoma.

Trial Name	Phase	Experimental Compounds	Setting
NCT03548571	II/III	Dendritic Cells transfected with mRNA from autologous tumor stem cells, survivin, and hTERT [95]	Primary treated patients with IDH wild-type, MGMT-promotor methylated GBM
NCT04277221	III	Autologous Dendritic Cell/Tumor Antigen (ADCTA-SSI-G1)	Recurrent GBM
NCT03149003	III	DSP-7888 Dosing Emulsion [102]	Recurrent or Progressive GBM (secondary GBMexcluded)
NCT02761070	III	Dose-dense temozolomide followed by Bevacizumab	Recurrent GBM
NCT02017717	III	Nivolumab +/− Ipilimumab	Recurrent GBM
NCT02667587	III	Nivolumab	Newly diagnosed MGMT-promotor methylated GBM (secondary GBMexcluded)
NCT00045968	III	DCVax-L [103]	Newly diagnosed GBM
NCT03025893	II/III	Sunitinib	Recurrent GBM

TME, tumor microenvironment; TERT, telomerase reverse transcriptase; MGMT, methyl Methylguanine-DNA Methyltransferase; GBM: Glioblastoma.

**Table 4:** Ongoing phase III trials targeting tumor microenvironment in other gliomas. TME, tumor microenvironment.

Trial Name	Phase	Experimental Compounds	Setting
NCT01236560	II/III	Bevacizumab	Newly diagnosed high-grade gliomas in young patients
NCT00045968	III	DCVax-L [103]	Newly diagnosed grade IV astrocytoma
NCT03149003	III	DSP-7888 Dosing Emulsion [102]	Grade 4 astrocytoma. Recurrent or Progressive disease.
NCT04532229	III	Nimotuzumab	Newly diagnosed diffuse intrinsic pontine glioma
NCT05009992	III	ONC201 [112]	Midline glioma

## Data Availability

The data presented in this study are available in this article.
